# Association of Diabetic Retinopathy and Arterial Stiffness With Cardiovascular Disease in Type 2 Diabetes

**DOI:** 10.7759/cureus.100396

**Published:** 2025-12-30

**Authors:** Chrysa Agapitou, Konstantinos Pappelis, Alexandros Chatzirallis, Stamatios Lampsas, Dimitrios Kazantzis, Alexia Risi-Koziona, Panagiotis Theodossiadis, Ignatios Ikonomidis, Vaia Lambadiari, Irini Chatziralli

**Affiliations:** 1 2nd Department of Ophthalmology, Attikon University Hospital, Medical School, National and Kapodistrian University of Athens, Athens, GRC; 2 2nd Department of Cardiology, Attikon University Hospital, Medical School, National and Kapodistrian University of Athens, Athens, GRC; 3 2nd Department of Internal Medicine, Research Institute and Diabetes Center, National and Kapodistrian University of Athens, Athens, GRC; 4 2nd Department of Ophthalmology, Medical School, National and Kapodistrian University of Athens, Athens, GRC

**Keywords:** arterial stiffness, cardiovascular disease, diabetic macular edema, diabetic retinopathy, endothelial dysfunction

## Abstract

Background: Diabetic retinopathy (DR) and diabetic macular edema (DME) are well-established microvascular complications of type 2 diabetes mellitus (T2DM), resulting from chronic hyperglycemia-induced damage to retinal vasculature that, in their advanced forms, are associated with an increased risk of cardiovascular disease (CVD).

Objective: This study aims to estimate the potential association between DR or DME and CVD, as well as systemic vascular metrics.

Methods: In a cross-sectional design study, 73 patients with T2DM were recruited. Demographic data and clinical characteristics of patients regarding their diabetes status and comorbidities were recorded. Patients underwent a thorough ocular examination, as well as endothelial function and arterial stiffness evaluation. Specifically, central systolic blood pressure (cSBP), central diastolic blood pressure (cDBP), pulse wave velocity (PWV), augmentation index (Aix), and the perfused boundary region of the sublingual arterial microvessels (PBR5-25) were measured. Multivariate binary logistic regression and mixed linear regression models were used to examine the associations between CVD, as well as systemic vascular metrics, and DR or DME.

Results: In a model adjusted for age, sex, BMI, smoking status, HbA1c levels, and hypertension, DR was significantly associated with higher odds for CVD (OR=13.3; 95% CI: 1.5-115.0, p=0.019). The presence of DME was not associated with CVD. Furthermore, higher PWV was significantly associated with more severe DR (b=0.09; 95% CI: 0.01-0.16, p=0.031).

Conclusions: There is a strong association between DR and CVD, while higher PWV was significantly associated with more severe DR. These findings suggest that screening for CVD is crucial in patients with DR, especially in cases of greater severity of DR.

## Introduction

Diabetes mellitus (DM) represents a significant global health concern, with estimates indicating that approximately 590 million individuals have the disease, while it is expected to climb sharply, reaching about 850 million by 2050 [1,2]. The rising prevalence of DM has been accompanied by an increase in the incidence of associated complications. As a prevalent microvascular complication, diabetic retinopathy (DR) affects an estimated 33% of individuals diagnosed with DM worldwide and represents the primary cause of vision loss in adults of working age [3]. With respect to macrovascular complications, individuals with DM exhibit an increased risk of developing cardiovascular diseases (CVD), encompassing atherosclerosis-related disorders such as coronary artery disease, peripheral arterial disease, and cerebrovascular disease [4]. These conditions represent the prominent causes of morbidity and mortality among the diabetic population [4]. It is worth noting that individuals with type 2 DM (T2DM) have a two- to four-fold elevated risk of developing coronary heart disease and ischemic stroke, along with a 1.5- to 3.6-fold increase in mortality, compared with non-diabetic individuals [5].

Previous studies have shown that DR, especially higher degrees of DR, was associated with high CVD incidence and all-cause mortality in patients with T2DM [6,7]. Barrot et al., in a large retrospective cohort study, reported that subjects with T2DM with any stage of DR had higher risks for coronary heart disease and major macrovascular events, except for stroke [8]. Additionally, the prospective case-control PRECISED study showed that the presence and degree of DR are independently associated with subclinical CVD in T2DM diabetic patients, suggesting a rationalized screening for coronary artery disease in T2DM based on prioritizing patients with DR, particularly those with a moderate-to-severe degree [9].

DR, diabetic macular edema (DME), and CVD are bound by overlapping pathophysiological processes in vascular function induced by hyperglycemia, including oxidative stress, chronic inflammation, and endothelial dysfunction, leading to microvascular injury in the retina and systemically [10]. These processes lead to thickening of the capillary basement membrane, loss of pericytes, and increased vascular permeability, giving rise to DR and DME, while simultaneously promoting atherosclerosis and endothelial dysfunction in the cardiovascular system [11]. Increased systemic inflammatory and oxidative stress markers, along with reduced nitric oxide bioavailability, have been reported in patients with DR and DME, indicating diffuse microvascular impairment beyond the retina [11]. In the retinal microvasculature, these processes lead to microaneurysms, capillary dropout, and blood-retinal barrier dysfunction, promoting vascular leakage and edema [11]. Similarly, microvascular alterations in the myocardium result in endothelial basement membrane thickening and endothelial dysfunction, leading to reduced tissue perfusion, elevated vascular resistance, and a prothrombotic state, thereby facilitating CVD progression in patients with T2DM [12]. Thus, microvascular impairment in the retina represents a marker of widespread vascular disease, reflecting the presence of similar pathogenic processes affecting the heart, brain, and other tissues [12].

Based on the above context, the primary objective of the present study was to confirm this relationship within a Greek population while integrating DR severity and DME with CVD and detailed systemic vascular function markers. Secondarily, we aimed to explore whether increasing DR severity is differentially associated with adverse systemic vascular profiles, potentially supporting a more integrated microvascular-macrovascular risk assessment framework.

## Materials and methods

A total of 73 patients with T2DM participated in this cross-sectional study. Recruitment took place at the 2nd Department of Ophthalmology, National and Kapodistrian University of Athens, Greece, from July 1, 2023, to December 31, 2024. Patients included in this study were human adults (aged 18 years or older), both males and females, and were diagnosed with T2DM and taking regular anti-diabetics. Patients with retinal disorders unrelated to DR, uncontrolled glaucoma (intraocular pressure >30 mmHg), uveitis, corneal or lens opacities, pregnancy, and systemic diseases (such as lupus, rheumatoid arthritis, and cancer) were excluded.

The study was conducted in accordance with the principles of the Declaration of Helsinki, and written informed consent was obtained from all participants. Ethical approval was granted by the Institutional Review Board of Attikon University General Hospital (Approval Code: 302/2021; Approval Date: June 2021). Demographic data, including age (years), gender (male/female), and smoking habits, were collected. Additionally, the following factors were recorded: antidiabetic treatment used, DM disease duration, comorbidities (hypertension, dyslipidemia), history of prior stroke or angioplasty, and occurrence of DM-related complications (DR, neuropathy, nephropathy, diabetic foot, CVD). Hypertension was defined as a documented diagnosis, use of antihypertensive medication, or office blood pressure ≥140/90 mmHg on at least two occasions, while dyslipidemia was defined as a documented diagnosis or use of lipid-lowering therapy.

Endothelial function was further assessed in all patients by measuring endothelial glycocalyx thickness. Specifically, an indirect assessment of endothelial glycocalyx integrity was performed by measuring the perfused boundary region (PBR) of the sublingual arterial microvessels with a diameter that ranged from 5 to 25 μm using Sidestream Dark Field imaging (Microscan, Glycocheck, Microvascular Health Solutions Inc., Salt Lake City, UT, USA) [13]. The camera was positioned sublingually, enabling the visualization of more than 3,000 microvascular segments. An elevated PBR indicates deeper penetration of red blood cells into the endothelial surface, reflecting a compromised glycocalyx with impaired barrier function, whereas a lower PBR suggests preserved glycocalyx integrity. Moreover, we measured carotid-femoral pulse wave velocity (PWV), the augmentation index (Aix), and central aortic pressures (central systolic and diastolic, cSBP and cDBP) using tonometry with the Complior (Alam Medical, Vincennes, France). According to the European Society of Hypertension (ESH) guidelines, PWV values exceeding 10 m/s in hypertensive patients indicate elevated arterial stiffness and reflect structural and functional alterations of the aortic wall. The Aix was defined as 100 × (P2 − P1)/PP, with P2 denoting the late backward systolic wave, P1 the early forward systolic wave, and PP representing pulse pressure, which quantifies the pressure increase resulting from reflected waves reaching the aorta. During systole, the volume of blood ejected into the aorta generates an initial pulse wave (early systolic peak, P1), which propagates along the aorta, reflects at the bifurcation, and gives rise to a secondary wave (late systolic peak, P2).

Regarding ophthalmic evaluation, best-corrected visual acuity (BCVA) was measured in all patients using Snellen charts, accompanied by a slit-lamp examination, dilated fundoscopy, and retinal imaging, including color fundus photography, optical coherence tomography (OCT) of the macular area and the optic nerve, as well as OCT-angiography (OCT-A) of the macula and optic nerve, using DRI Triton (Topcon), as previously described [14]. All imaging scans were conducted by a single experienced operator (CA). Fundus fluorescein angiography (FFA) using Spectralis (Spectralis HRA+OCT, Heidelberg Engineering, Germany) was performed at the discretion of the attending physician to detect or rule out neovascularization. DR was classified per the International Clinical Diabetic Retinopathy Disease Severity Scale as no DR, mild, moderate, or severe non-proliferative DR (NPDR), and proliferative DR (PDR). DME was also assessed. The primary objective was to explore significant relationships between DR/DME and CVD, as well as endothelial and hemodynamic parameters, including PBR5-25, cSBP, cDBP, PWV, and Aix.

Patient characteristics were summarized using mean ± standard deviation (SD) for continuous variables, whereas categorical variables were expressed as frequencies and percentages. The Kolmogorov-Smirnov normality test was applied to test the normality of the distribution. We used multivariable binary logistic regression mixed models, accounting for inter-eye correlations, to examine the association between CVD and DR or DME in order to account for the correlation between eyes from the same participant. We also used multivariable binary logistic regression and mixed-effects linear regression models to examine the association between DR outcomes and systemic vascular metrics (PBR5-25, cSBP, cDBP, PWV, and Aix). All models were adjusted for age, sex, body mass index (BMI), smoking status, HbA1c (%), and arterial hypertension. We calculated the corresponding odds ratios (ORs) and beta coefficients with the 95% confidence intervals (CIs). A p-value ≤ 0.05 was considered statistically significant. All analyses were performed with IBM SPSS Statistics for Windows, Version 29 (Released 2023; IBM Corp., Armonk, New York).

## Results

A total of 73 subjects were included. Table 1 presents the demographic and clinical features of the study population. The mean age of patients was 66.3±8.4 years; 58.9% (n=43) of 73 patients were male, and 41.1% (n=30) were female. The mean BMI was 30.3±5.1 kg/m^2^. The average duration of DM was 16 ± 10.7 years. The mean HbA1c was 7.3 ± 1.1%. Regarding comorbidities and DR complications, 75.3% (n=55) of 73 patients had hypertension, 79.5% (n=58) had dyslipidemia, 10.9% (n=8) had nephropathy, 24.7% (n=18) had neuropathy, 8.2% (n=6) had diabetic foot, and 23.3% (n=17) had CVD. In addition, 8.2% (n=6) of patients had a previous stroke, and 15.1% (n=11) of patients had undergone angioplasty. As far as the DR stage is concerned, 27.4% (n=20) of patients presented no DR, 6.9% (n=5) mild NPDR, 17.8% (n=13) moderate NPDR, 35.6% (n=26) severe NPDR, and 12.3% (n=9) PDR, while DME was present in 41.4% (n=30) of patients.

**Table 1:** Demographic and clinical characteristics of the study sample (n=73 patients). DME: diabetic macular edema; DR: diabetic retinopathy; NPDR: non-proliferative diabetic retinopathy; PDR: proliferative diabetic retinopathy; PBR: perfused boundary region

Variable	Value
Age (mean ± SD, years)	66.3 ± 8.4
Male sex, female sex (n, %)	43 (58.9%), 30 (41.1%)
Weight (mean ± SD, kg)	86.5 ± 15.4
Height (mean ± SD, cm)	169.1 ± 9.4
Body Mass Index (mean ± SD, kg/m^2^)	30.3 ± 5.1
Smoking (n, %)	29 (39.7%)
Diabetes mellitus duration (mean ± SD, years)	16.0 ± 10.7
HbA1c (mean ± SD, %)	7.3 ± 1.1
Hypertension (n, %)	55 (75.3%)
Dyslipidemia (n, %)	58 (79.5%)
Nephropathy (n, %)	8 (11.0%)
Neuropathy (n, %)	18 (24.7%)
Diabetic Foot (n, %)	6 (8.2%)
Cardiovascular disease (n, %)	17 (23.3%)
Stroke (n, %)	6 (8.2%)
Angioplasty (n, %)	11 (15.1%)
PBR_5-25_ (mean ± SD, μm)	2.2 ± 0.3
Central Systolic Blood Pressure (mean ± SD, mmHg)	130.3 ± 19.6
Central Diastolic Blood Pressure (mean ± SD, mmHg)	78.4 ± 9.8
Augmentation Index (mean ± SD, %)	16.7 ± 22.3
Pulse Wave Velocity (mean ± SD, m/s)	18.5 ± 19.8
Diabetes current treatment (n, %)	
Insulin	38 (52.1%)
Metformin	50 (68.5%)
Sulfonylurias	6 (8.2%)
GLP-1RA	24 (32.9%)
SGLT-2	28 (38.4%)
DPP-4	18 (24.7%)
Diabetic retinopathy stage (n, %)	
No DR (n, %)	20 (27.4%)
Mild NPDR (n, %)	5 (6.9%)
Moderate NPDR (n, %)	13 (17.8%)
Severe NPDR (n, %)	26 (35.6%)
PDR (n, %)	9 (12.3%)
DME (n, %)	30 (41.1%)

Table 2 shows the results of multivariate analysis regarding the association between CVD and DR or DME. After adjusting for age, sex, BMI, smoking status, HbA1c levels, and hypertension, DR was significantly associated with higher odds for CVD (OR=13.3; 95% CI: 1.5-115.0, p=0.019). Accordingly, patients with both NPDR and PDR were associated with significantly higher odds for CVD compared to patients without DR (OR=19.9; 95% CI: 1.4-280.4 for NPDR and OR=12.2; 95% CI: 1.3-111.1 for PDR, respectively, p=0.027). The presence of DME was not associated with CVD.

**Table 2:** Results of the multivariate analysis regarding the association between cardiovascular disease and diabetic retinopathy. *: significant at the 0.05 level. All models were adjusted for age, sex, body mass index, smoking status, HbA1c (%), and arterial hypertension. DR (Reference = no DR); DR stage (Reference = no DR); DME (Reference = no DME). DR: diabetic retinopathy; OR: odds ratio; CI: confidence intervals; DME: diabetic macular edema

Variable		OR (95% CI)	P-value
Model 1: DR (yes/no)			
DR		13.3 (1.5 to 115.0)	0.019*
Model 2: DR stage			
NPDR	19.9 (1.4 to 280.4)		0.027*
PDR	12.2 (1.3 to 111.1)		
Model 3: DME (yes/no)			
DME	1.0 (0.99 to 1.01)		1.0

Tables 3-5 show the results of the multivariate analysis regarding the association between DR and systemic vascular metrics. Using a model adjusted for potential confounders, including age, sex, BMI, smoking status, HbA1c, and hypertension, higher PWV was significantly associated with more severe DR (b=0.09; 95% CI: 0.01 to 0.16; p=0.031), and a similar trend was noted for higher PWV and presence of DR (OR=1.22; 95% CI: 0.98 to 1.51; p=0.071). No other measured systemic parameter was associated with DR or DME.

**Table 3:** Results of the multivariate analysis regarding the association between the presence of diabetic retinopathy (yes/no) and systemic vascular metrics. *: significant at the 0.05 level. All models were adjusted for age, sex, body mass index, smoking status, HbA1c (%), and arterial hypertension. DR: diabetic retinopathy; OR: odds ratio; CI: confidence intervals; PBR 5-25: perfused boundary region at 5-25 nm; Aix: augmentation index; PWV: pulse wave velocity; cSBP: central systolic blood pressure; cDBP: central diastolic blood pressure

Parameter	OR (95% CI)	P-value
PBR_5-25_ (μm)	3.0 (0.1 to 70.2)	0.50
Aix	1.01 (0.95 to 1.07)	0.80
PWV (m/s)	1.22 (0.98 to 1.51)	0.071
cSBP (mmHg)	1.02 (0.97 to 1.07)	0.49
cDBP (mmHg)	1.00 (0.1 to 8.9)	0.97

**Table 4:** Results of the multivariate analysis regarding the association between the diabetic retinopathy stage and systemic vascular metrics. *: significant at the 0.05 level. All models were adjusted for age, sex, body mass index, smoking status, HbA1c (%), and arterial hypertension. DR: diabetic retinopathy; OR: odds ratio; CI: confidence intervals; PBR 525: perfused boundary region at 525 nm; Aix: augmentation index; PWV: pulse wave velocity; cSBP: central systolic blood pressure; cDBP: central diastolic blood pressure

Parameter	OR (95% CI)	P-value
PBR_5-25_ (μm)	0.66 (-0.64 to 1.96)	0.31
Aix	0.00 (-0.02 to 0.03)	0.85
PWV (m/s)	0.09 (0.01 to 0.16)	0.031*
cSBP (mmHg)	0.01 (-0.01 to 0.03)	0.48
cDBP (mmHg)	0.00 (-0.04 to 0.04)	0.84

**Table 5:** Results of the multivariate analysis regarding the association between diabetic macular edema and systemic vascular metrics. *: significant at the 0.05 level. All models were adjusted for age, sex, body mass index, smoking status, HbA1c (%), and arterial hypertension. DR: diabetic retinopathy; OR: odds ratio; CI: confidence intervals; PBR 525: perfused boundary region at 525 nm; Aix: augmentation index; PWV: pulse wave velocity; cSBP: central systolic blood pressure; cDBP: central diastolic blood pressure; DME: diabetic macular edema

Parameter	OR (95% CI)	P-value
PBR_5-25_ (μm)	0.9 (0.1 to 6.4)	0.91
Aix	1.00 (0.96 to 1.04)	0.96
PWV (m/s)	1.0 (0.9 to 1.2)	0.58
cSBP (mmHg)	1.01 (0.98 to 1.04)	0.53
cDBP (mmHg)	1.00 (0.94 to 1.07)	0.91

## Discussion

The principal message of the study is that DR, either NPDR or PDR, was associated with higher odds for CVD, whereas DME was not observed to be associated with CVD in this study. In addition, exploratory analyses suggested that increased PWV was associated with greater DR severity. Given the relatively small number of participants with DR and CVD events, as well as the cross-sectional design of the study, these findings should be interpreted with caution and warrant confirmation in larger prospective studies.

Our study supports the previously reported association between DR and an increased risk of CVD, observed in a Greek population attending a tertiary referral center. Numerous epidemiological studies have demonstrated a significant correlation between the presence as well as severity of DR and the incidence of major cardiovascular events, including coronary artery disease, myocardial infarction, and stroke [6-8]. Interestingly, Castelblanco et al. found that the presence of DR was a strong predictor of CVD independently of the presence of subclinical carotid atherosclerosis in subjects with T2DM without previous clinical CVD, suggesting a solid link between DR and CVD in such patients [15].

The association between microvascular complications and CVD is increasingly supported by recent experimental and clinical findings [16]. The underlying pathophysiological mechanisms linking DR and CVD are believed to share common risk factors, such as chronic hyperglycemia, hypertension, dyslipidemia, and systemic inflammation, all of which contribute to both microvascular and macrovascular damage [15]. There may be several pathophysiological parallels between microvascular and macrovascular complications. Hyperglycemia has been proposed to induce pathways, including excessive mitochondrial superoxide generation, polyol, hexosamine, and protein kinase C activation, as well as advanced glycation end-product formation. Such biochemical effects induce intracellular oxidative stress, upregulation of inflammatory mediators, cardiomyocyte dysfunction, and vascular damage, thereby driving the development of micro- and macrovascular complications [17].

On the other hand, PWV is a well-established, non-invasive measure of arterial stiffness and an independent predictor of cardiovascular morbidity and mortality. Several studies have demonstrated a significant association between the severity of DR and elevated PWV, suggesting that progression of retinal microvascular damage may parallel systemic vascular dysfunction [18-22]. Cardoso et al. found a stepwise increase in PWV in more severe stages of DR in patients with T2DM, suggesting a dose-response association between retinal microvascular damage and systemic arterial stiffness [18]. Accordingly, Liu et al. reported that an increasing degree of PWV exhibited a positive relationship with the severity of DR, with elevated levels being strongly associated with the likelihood of severe DR, notably PDR [19]. Notably, the association between DR and PWV may reflect the effects of altered intravascular hemodynamics [20]. In fact, arterial stiffness itself may impair retinal perfusion, potentially exacerbating DR [20]. Our recent study provides additional evidence supporting a potential relationship, since we found that increased PWV was associated with enlargement of the foveal avascular zone (FAZ) area and greater FAZ perimeter, which are considered both biomarkers of more severe DR and damaged microvascular retinal circulation [23].

Moreover, DR staging may serve as a surrogate marker of systemic vascular health, reflecting the cumulative burden of microvascular and macrovascular dysfunction, given its association with endothelial damage, chronic inflammation, and atherosclerotic progression [24,25]. Particularly, early DR staging (mild and moderate NPDR) has been linked to a higher risk of microalbuminuria, while macroalbuminuria was associated only with those patients who had either severe NPDR, very severe NPDR, or PDR [26]. Stroke and other cerebrovascular diseases are also vascular complications whose association and risk with DR have a "dose-dependent" relationship, where greater severity of DR is linked to a progressively higher risk of CVD [27]. Moreover, WHO-MSVDD and other studies show that the risk of coronary heart disease is graded with a dose-dependent relationship with the severity of the DR stage [28,29]. Finally, according to WESDR data, the presence of severe NPDR or PDR was associated with a higher risk of subsequent lower limb amputation when contrasted with individuals showing absent or mild retinopathy [30]. Overall, the severity of DR staging appears to be a powerful and dose-dependent marker for the risk of a wide range of systemic vascular complications.

The cross-sectional nature of the study represents a possible limitation, which does not allow the confirmation of a causal association between DR and CVD, as well as the small sample size. In addition, the quantification of the endothelial glycocalyx in the retinal microcirculation in vivo is indirect, derived approximately from the sublingual microcirculation [23]. However, the data used were based on active screening and have great reliability. Moreover, data on the duration and adequacy of hypertension and dyslipidemia control were not systematically collected despite the importance of vascular outcomes' evaluation. Given the cross-sectional design and modest sample size, our findings should be interpreted as exploratory and hypothesis-generating, highlighting associations rather than definitive causal relationships, and warranting confirmation in larger, longitudinal studies.

## Conclusions

In conclusion, this study showed a strong association between DR and CVD, as well as between DR and PWV, suggesting that the presence of DR may be indicative of an increased risk for CVD. Given this evidence, DR staging may serve as a clinically accessible surrogate marker for systemic vascular health, while PWV may offer complementary prognostic information in patients with T2DM. Together, these parameters could aid in the early identification of individuals at increased risk for CVD and guide more comprehensive vascular risk management strategies. However, given the cross-sectional design and modest sample size, causal relationships cannot be established, and the results should be interpreted as exploratory. Future longitudinal studies are needed to confirm whether DR and PWV can reliably contribute to cardiovascular risk assessment in this population.
